# Ethnic and Sex Differences of White Matter Tracts in Aging

**DOI:** 10.1002/alz70856_106887

**Published:** 2026-01-08

**Authors:** Flavio Carhuavilca, Merike Lang, Alicia Goytizolo, Sebastian Garcia, Adina Patrascanu, Ranjan Duara, Warren W Barker, Idaly Velez‐Uribe, Glenn E. Smith, Stephen A. Coombes, Monica Rosselli

**Affiliations:** ^1^ Florida Atlantic University, Davie, FL, USA; ^2^ Wien Center for Alzheimer's Disease and Memory Disorders, Miami Beach, FL, USA; ^3^ Mount Sinai Medical Center, Miami Beach, FL, USA; ^4^ 1Florida Alzheimer's Disease Research Center, Miami, FL, USA; ^5^ Florida International University, Miami, FL, USA; ^6^ University of Miami School of Medicine, Miami, FL, USA; ^7^ University of Florida, Gainesville, FL, USA; ^8^ Wien Center for Alzheimer's Disease Research Center, Miami Beach, FL, USA; ^9^ Mayo Clinic, Rochester, MN, USA; ^10^ Wien Center for Alzheimer's Disease and Memory Disorders, Miami Beach, FL, USA

## Abstract

**Background:**

Research on gender and ethnic differences in white matter tracts yields mixed results. Although men show larger global corpus callosum (CC) in absolute values, women have demonstrated larger splenium and isthmus. Additionally, men have shown greater white matter and fractional anisotropy (FA) in multiple regions than women. This study investigates sex and ethnic differences in the corpus callosum and transcallosal white matter tissue microstructure of older individuals with normal cognition and memory disorders.

**Method:**

191 participants (Female *n* = 120; Hispanics *n* = 115; Non‐Hispanics n = 76; Age mean = 71.07; SD = 7.75) were included in the sample from the 1Florida ADRC with cognitively normal (*n* = 75), MCI (*n* = 88), and dementia (*n* = 28) diagnoses. Corpus callosum volumes were collected using a Siemens Skyra 3T MRI scanner and were divided into anterior, mid‐anterior, central, mid‐posterior, and posterior regions. Also, MRI‐based free water diffusion tensor imaging (FW‐DTI) measured FW‐corrected FA (fwcFA) to assess and compare the integrity of transcallosal tract templates (TCATT) across motor, temporal, parietal, and occipital regions across males and females and ethnic groups.

**Result:**

Univariate ANCOVAs controlling for age and clinical diagnosis were performed; females had larger normalized CC volumes in the posterior, *F*(1, 184) = 8.92, *p* = .003, *η_p_^2^* = .046, mid‐anterior, *F*(1,184)= 5.68, *p* = .018, *η*_p_^2^ = .030, and anterior, *F*(1,184)= 14.54, *p* < .001, *η*_p_^2^ = .073, regions, which all survived FDR corrections, with no ethnic differences observed. For transcallosal tracts, females had higher fwcFA in the inferior temporal gyrus, *η*_p_^2^ Hispanics had higher fwcFA in the premotor cortex, *η*_p_^2^ *η*_p_^2^

**Conclusion:**

Sex and ethnicity influence white matter changes during aging independently of age and cognitive diagnosis. However, the effect explained by these demographic variables was very small, suggesting the influence of additional factors.

